# *In silico* biotechnological potential of *Bacillus* sp. strain MHSD_37 bacterial endophyte

**DOI:** 10.1186/s12864-024-10305-2

**Published:** 2024-04-24

**Authors:** Pfariso Maumela, Adivhaho Khwathisi, Ntakadzeni Edwin Madala, Mahloro Hope Serepa-Dlamini

**Affiliations:** 1 Department of Biotechnology and Food Technology, Faculty of Science, University of Johannesburg, Doornfontein Campus, 2028 Johannesburg, P.O. Box 17011, South Africa; 2 Department of Biochemistry and Microbiology, Faculty of Science, Engineering and Agriculture, University of Venda, Private Bag X5050, 0950 Thohoyandou, South Africa

**Keywords:** Bioremediation, Biofertilization, Biocontrol, Bio-nanotechnology, Bacterial endophytes

## Abstract

**Background:**

Endophytic bacteria possess a range of unique characteristics that enable them to successfully interact with their host and survive in adverse environments. This study employed *in silico* analysis to identify genes, from *Bacillus* sp. strain MHSD_37, with potential biotechnological applications.

**Results:**

The strain presented several endophytic lifestyle genes which encode for motility, quorum sensing, stress response, desiccation tolerance and root colonisation. The presence of plant growth promoting genes such as those involved in nitrogen fixation, nitrate assimilation, siderophores synthesis, seed germination and promotion of root nodule symbionts, was detected. Strain MHSD_37 also possessed genes involved in insect virulence and evasion of defence system. The genome analysis also identified the presence of genes involved in heavy metal tolerance, xenobiotic resistance, and the synthesis of siderophores involved in heavy metal tolerance. Furthermore, LC-MS analysis of the excretome identified secondary metabolites with biological activities such as anti-cancer, antimicrobial and applications as surfactants.

**Conclusions:**

Strain MHSD_37 thereby demonstrated potential biotechnological application in bioremediation, biofertilisation and biocontrol. Moreover, the strain presented genes encoding products with potential novel application in bio-nanotechnology and pharmaceuticals.

## Background

*Solanum nigrum*, commonly known as black nightshade, is a medicinal plant native to Eurasia and has been introduced to South Africa, America, and Australasia [1]. *S. nigrum* is traditionally used as a medicine for ailments including tooth ache, tonsilitis, abdominal pain, fever, tumor, and inflammation [2]. *S. nigrum* can grow in heavy metal contaminated soil and water and has been reported to be an excellent hyperaccumulator of cadmium (Cd) [3]. The phytoremediation potential of *S. nigrum* is enhanced through their association with bacteria and fungal microbes, commonly knows as endophytes [4–5]. The plant thus makes an important source for the prospecting of microbial endophytes with potential biotechnological applications [3, 5].

Bacterial endophytes (BEs) have symbiotic relationships with their host plants [4] and are capable of alleviating metal phytotoxicity through the biotransformation of metal ions to non-toxic forms, precipitation of metal ions, or intracellular accumulation [5–6]. BEs synthesize metallothionein-like proteins and siderophores which bind to and immobilize heavy metals [7]. Improved plant tolerance to heavy metals stress and their immobilization are reportedly linked to an increased production of organic acids including oxalic acid, acetic acid, tartaric acid, succinic acid, and formic acid [8–9]. Organic acids play a significant role in the complexation of toxic metals during bioremediation and enhance the solubility and uptake of essential metal ions by plants [10]. BEs produce siderophores such as pyoverdine, pyochelin and alcaligin E which have been illustrated to mobilize heavy metals such as lead (Pb) and chromium (Cr) thereby enhancing their uptake by plants [11–12].

BEs like the fungal counterparts colonize plant tissue and synthesize biological products that promote plant health, growth, and development for their host [13]. Luo and coworkers [14] reported a *Bacillus* sp. capable of producing indoleacetic acid (IAA), siderophores and 1-aminocyclopropane-1-carboxylate deaminase (ACC) when inoculated to sweet sorghum growing in Cd contaminated soils. The siderophores were responsible for the increase in the aerial biomass and improved root development as well as plant growth [14]. Moreover, BEs synthesize and secrete metabolites with antimicrobial activity and consequently play a significant role in disease prevention in their host [15]. BEs protect host plants from herbivorous pests through physical and chemical modification of the leaves by altering the lamina density and cellulose content [16].

The current study reports on the draft genome sequence of a bacterial endophytes *Bacillus* sp. MHSD_37 previously isolated from *S. nigrum*. The whole genome sequence (WGS) analysis revealed significant genes involved in the symbiont relationship between the *Bacillus* species and its host. Genes involved in heavy metal detoxification, encoding for plant pest virulence factors, and the synthesis of plant growth promoting factors were identified as important factors for the potential biotechnological application of *Bacillus* sp. in bioremediation, biocontrol and biofertilization, respectively.

## Results

### Basic genomic characteristics of the strain

The *de novo* assembly of *Bacillus* sp. strain MHSD_37 (BioProject ID: PRJNA1010788) sequence resulted in a genome size of 5 139 594 bp composed of 43 contigs and a G + C content of 35.3% (Table 1). The PGAP annotation identified a total of 75 tRNA, 1 tmRNA and 94 rRNA, and 5 242 protein coding sequences (CDS). The CRISPRFinder analysis identified four probable CRISPR repeat regions located on the chromosome (Table 1). The detected CRISPR sequences belong to the III-A, III-B, IV-A and IV-B subtypes. A single potential intact phage (Table 1) was identified in strain MHSD_37 using PHASTER. The region was located between positions 1 450 718 and 1 520 653, with a length of 69.9 kb. Furthermore, the region had a G + C content of 35% (Table 1), and a G + C content difference of 0.35% compared to the chromosome of *Bacillus* sp. strain MHSD_37. Interestingly the phage region of strain MHSD_37 was comparable to those of closely related strains, *Bacillus paranthracis* MCCC 1A00395 with a region size of 83 kb region and a G + C content of 36%, and *Bacillus tropicus* N24 with a region of 58 kb and a G + C content of 35%. The PHASTER analysis thus indicate that the region represents a potential phage.

**Table 1:** Genome characteristic for *Bacillus* sp. strain MHSD_37.

Genome characteristics	Value
Total size (bp)	5,139,851
Contigs	43
% G + C content	35.31
Genes (total)	5,375
CDSs (total)	5,289
Genes (coding)	5,118
CDSs (with protein)	5,118
Genes (RNA)	86
rRNAs	3, 2, 1 (5 S, 16 S, 23 S)
complete rRNAs	3 (5 S)
partial rRNAs	2, 1 (16 S, 23 S)
tRNAs	75
ncRNAs	5
Pseudo Genes (total)	171
CDSs (without protein)	171
CRISPR repeat regions	4
Position of phage region	1,450,718-1,520,653
Size of phage region (kb)	69.9
Phage region G + C content (%)	35

### Phylogenetic analysis

**Fig. 1:**
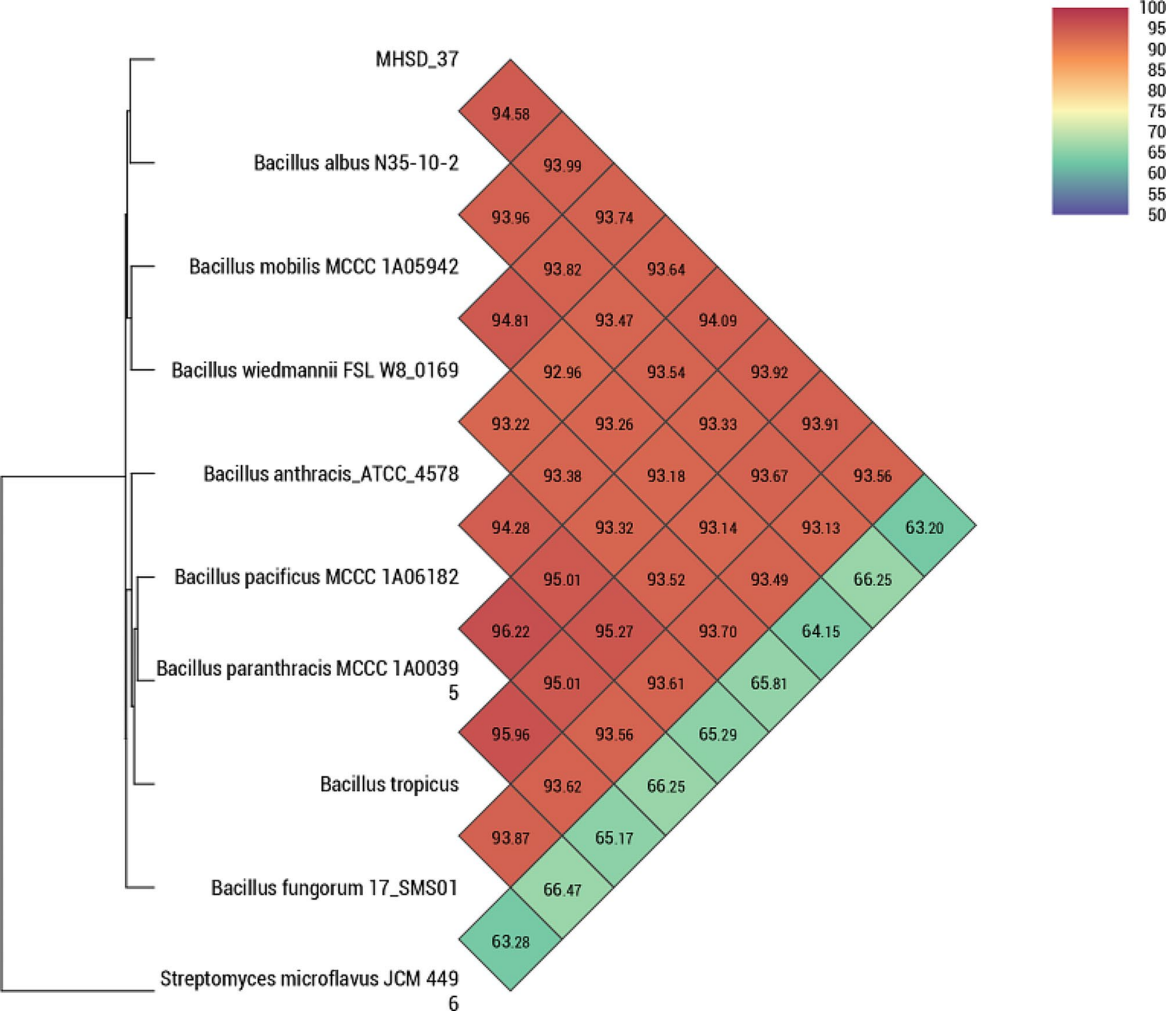
Heat-map, based on ANI values, for *Bacillus* sp. strain MHSD_37 and related species.

The TYGS was used to determine the phylogenomic relationships and identification of strain MHSD_37. The whole genome based phylogenetic analysis showed that strain MHSD_37 was closely related to *Bacillus albus* strain N35-10-2^T^ with a digital DNA-DNA hybridization (dDDH) of 58%, which was the highest observed dDDH with a closely related species (Table 2). In contrast the ANI analysis revealed that strain MHSD_37 was closest to *B. paranthracis* MCCC 1A00395 with a value of 96.2% (Fig. 1), which was above the species boundary value ANI > 95–96% (Fig. 1) [26]. Moreover, the strain had an ANI value of 95.9 with *B. tropicus*, which was also above the species boundary value [26]. On the other hand, the ANI value between strain MHSD_37 and *Bacillus albus* strain N35-10-2^T^ was 94.5%.

**Table 2:** Pairwise comparisons between *Bacillus* sp. MHSD_37 and related species.

Subject strain	dDDH (d0, in %)	C.I. (d0, in %)	dDDH (d4, in %)	C.I. (d4, in %)	dDDH (d6, in %)	C.I. (d6, in %)	G + C content difference (in %)	G + C content (%
*Streptomyces microflavus* JCM 4496	12.5	[9.8–15.8]	63.4	[60.4–66.2]	12.9	[10.6–15.7]	35.92	71.23
*Bacillus albus* N35-10-2	67.8	[63.9–71.4]	58.2	[55.4–61.0]	67.8	[64.3–71.0]	0.38	35.69
*Bacillus pacificus* MCCC 1A06182	74.6	[70.6–78.2]	55.2	[52.5–57.9]	72.8	[69.3–76.0]	0.11	35.42
*Bacillus paranthracis* MCCC 1A00395	75	[71.0–78.6]	54.2	[51.5–56.9]	72.8	[69.3–76.0]	0.13	35.44
*Bacillus tropicus* N24	77.3	[73.3–80.8]	53.6	[50.9–56.3]	74.5	[71.0–77.7]	0.11	35.42
*Bacillus mobilis* MCCC 1A05942	67.1	[63.2–70.7]	53.6	[50.9–56.3]	66	[62.6–69.2]	0.04	35.35
*Bacillus wiedmannii* FSL W8-0169	74.4	[70.4–78.0]	53.3	[50.6–55.9]	72	[68.6–75.3]	0.12	35.43
*Bacillus fungorum* 17-SMS-01	60	[56.3–63.5]	52.7	[50.0–55.3]	59.6	[56.4–62.8]	0.34	35.65
*Bacillus anthracis* ATCC 14,578	72.8	[68.8–76.4]	52.5	[49.8–55.1]	70.4	[67.0–73.7]	0.07	35.38
*Bacillus cereus* ATCC 14,579	73.4	[69.4–77.0]	43.8	[41.2–46.3]	67.9	[64.4–71.1]	0.03	35.34
*Bacillus luti* MCCC 1A00359	67.7	[63.8–71.3]	43.7	[41.1–46.2]	63.2	[59.9–66.5]	0.13	35.44
*Bacillus thuringiensis* ATCC 10,792	61.4	[57.7–64.9]	43.5	[41.0–46.1]	58.1	[54.9–61.2]	0.49	35.8
*Bacillus toyonensis* NCIMB 14,858	67.1	[63.2–70.7]	42.3	[39.8–44.9]	62.3	[59.0–65.5]	0.24	35.55
*Bacillus paramycoides* NH24A2	55.8	[52.2–59.3]	36.8	[34.3–39.3]	51.2	[48.2–54.3]	0.12	35.43

### Endophytic genes characterization

**Fig. 2:**
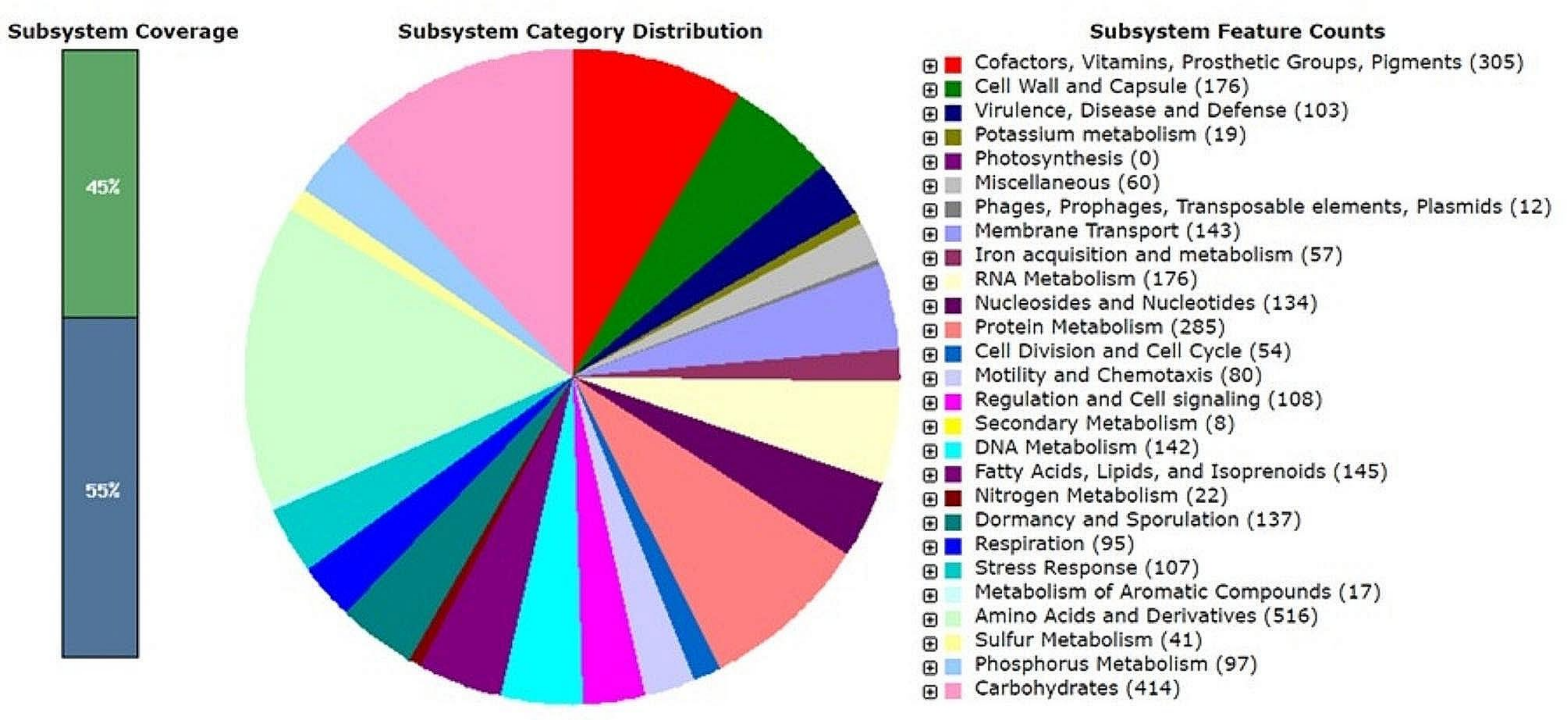
RAST annotation for the *Bacillus* sp. strain MHSD_37 genome.

The genome annotation and functional classification of the genes in *Bacillus* sp. strain MHSD_37, based on RAST annotation (Fig. 2), predicted an array of putative genes important for the endophytic lifestyle. The genome annotation data (Table 3) confirmed the presence of several traits related to the endophytic lifestyle of strain MHSD_37. The analysis of strain MHSD_37 genome identified putative genes involved in carbohydrate metabolism, mobilization as well as uptake of nutrients such as iron, nitrogen, and phosphate, motility, cell adhesion, membrane transport proteins, secretion and delivery systems, stress tolerance, detoxification, host cell wall modification, and transcriptional regulators (Table 3).

**Table 3:** Endophytic characteristics of *Bacillus* sp. MHSD_37.

Description	Role
Sensor histidine kinase	Early phytopathogen detection
Two-component sensor kinase YvcQ	Early phytopathogen detection
Biotin	Root colonisation by symbionts
Thiamine	Pathogenesis
Tyrosine-protein kinase	EPS production/stress response and pathogen attack
Capsule polysaccharide	Protection against toxic compounds and desiccation
Techoic acid	Protection from various threats and adverse conditions
Polysaccharide deacetylases	Bacterial evasion of lyzozyme
Xylanase chitin deacetylase	Protection from pest secreted chitinase/pest biocontrol
Sortase	Bacterial adhesion, biofilm formation, and immune escape
EPS	Biofilm production and Food/pharmaceutical applications
Superoxide dismutase	Nuetralise ROS
Manganese superoxide dismutase	Nuetralise ROS
Methylenetetrahydrofolate dehydrogenase	Viral resistance
Flagellin protein FlaA	Immune stimulator
N-acyl homoserine lactone hydrolase	Quorum sensing
Autoinducer 2 (AI-2) aldolase LsrF (EC 4.2.1.-)	Quorum sensing
Autoinducer 2 (AI-2) modifying protein LsrG	Quorum sensing
S-ribosylhomocysteine lyase (EC 4.4.1.21)	Quorum sensing
LysR family transcriptional regulator	Regulate QS and

Plant colonization is a result of a combination of factors including quorum sensing (QS), bacterial attachment and motility, neutralizing competition, and nutrient acquisition. The analysis identified a gene coding for N-acyl homoserine lactone hydrolase (Table 3), which plays a role in quorum quenching through the degradation of N-acyl homoserine lactone. Three genes involved in the synthesis of autoinducer-2 (AI-2), a universal mediator of inter- and intraspecies quorum sensing in bacteria, were identified. The genes were *lux*S, *lsr*F, and *lsr*G, coding for S-ribosylhomocysteine lyase (EC 4.4.1.21), autoinducer 2 (AI-2) aldolase (EC 4.2.1.-) and autoinducer 2 (AI-2) modifying protein LsrG, respectively.

Moreover, the analysis identified the genes encoding for *Lys*R family transcriptional regulator, a key regulator of genes involved in QS, metabolism, virulence, and motility. The presence of superoxide and manganese superoxide dismutase related genes was identified. The genes are involved in the detoxification of reactive oxygen species (ROS) which enables endophytes to evade the host defense system. Furthermore, the genes encoding for sensor histidine kinase and two-component sensor kinase *Yvc*Q, involved in phytopathogen detection, were also identified from the genome of strain MHSD_37.

**Fig. 3:**
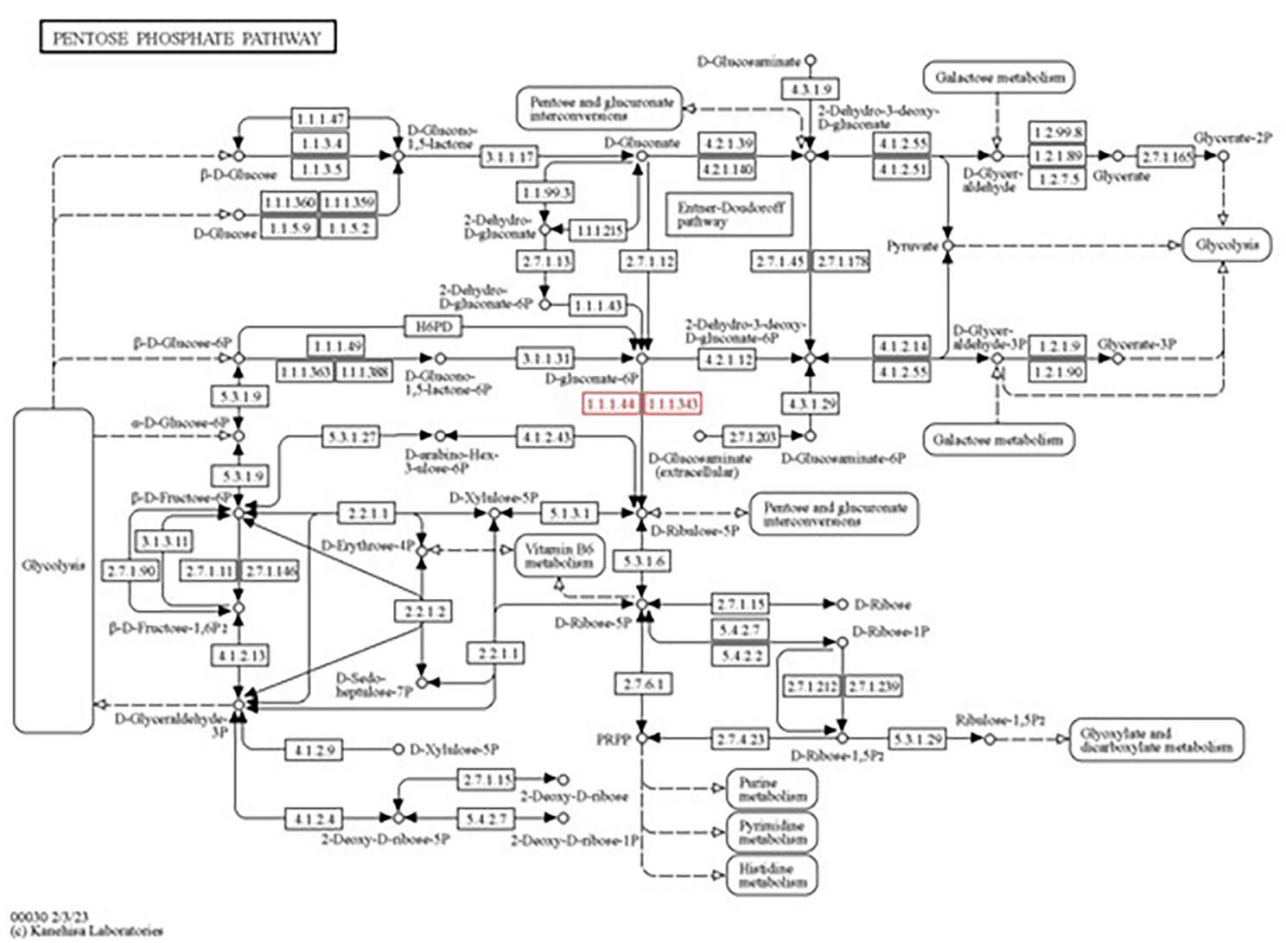
KEGG mapping of genes involved in the pentose phosphate pathway. The elements highlighted in red were identified from the genome of strain MHSD_37.

Carbohydrate metabolism is central for the energy production and consequently for the endophytes to sustain life and reproduction. The strain MHSD_37 encodes for two genes, ribose 5-phosphate isomerase and 6-phosphogluconate dehydrogenase (Table 3), found in the pentose phosphate pathway, EC 5.3. 1.6 and 1.1.1.44, respectively (Fig. 3). Moreover, the ability of endophytes to degrade plant polymers is an important carbohydrate metabolism pathway because it enables the use of abundant plant polymers such as starch as a carbon substrate. Strain MHSD_37 possessed two genes, encoding for oligo-1,6-glucosidase and alpha-glucosidase, involved in starch metabolism.

### Biotechnology potential characteristics

The genome analysis revealed that strain MHSD_37 had a range of genes involved in plant growth promotion, detoxification of toxic heavy metals as well as hydrocarbons, and virulence against plant pest which are detailed below.

### Plant growth promotion

The genome of strain MHSD_37 comprised of genes which are involved in nitrogen fixation, nitrate, and sulphur assimilation as well as phosphate solubilization (Table 4). Four genes involved in nitrogen fixation, namely Fe-S cluster assembly protein SufB, Fe-S cluster assembly ATPase SufC, Fe-S cluster assembly protein SufD, and Folate-dependent protein for Fe-S cluster synthesis/repair in oxidative stress, were identified from the genome of strain MHSD_37. In addition, the genome contains biosynthetic genes for exopolyphosphatase *(ppx*), secreted alkaline phosphatase (*sap*), and alkaline phosphatase synthesis transcriptional regulatory protein (*pho*P), responsible for phosphate solubilization.

**Table 4:** A summary of the biotechnological potential of MHSD_37.

Biotechnological potential	Description	Significance/role
Vaccine development	Bifunctional metallophosphatase/5’-nucleotidase	
Bionanotechnology	Ferritin-like protein 2	Metalloprotein
Biopesticide	internalin, putative	Virulence factor
	LSU ribosomal protein L35p	Virulence factor
	Quinolinate synthetase (EC 2.5.1.72)	Virulence factor
	Osmosensitive K + channel histidine kinase KdpD/E	Virulence factor
	Glutathione-regulated potassium-efflux system protein KefKL	Virulence factor
	Trk system potassium uptake protein TrkA	Virulence factor
	HtrA protease/chaperone protein	Defence system regulator
	Serine/threonine protein kinases	Defence system regulator
	Kynureninase	Defence system regulator
Bioremediation	Cobalt-zinc-cadmium resistance protein CzcA	Metal resistance
	Cobalt-zinc-cadmium resistance protein CzcC	Metal resistance
	Cobalt-zinc-cadmium resistance protein CzcD	Metal resistance
	Arsenical-resistance protein	Metal resistance
	Arsenical resistance operon repressor	Metal resistance
	Copper tolerance protein	Metal tolerance
	Zn(II) and Co(II) transmembrane diffusion facilitator	Metal tolerance/resistance by efflux of ions
	Large-conductance mechanosensitive channel	Osmotic stress
	Petrobactin	Siderophore
	Glutathione S-transferase family protein	Xenobiotic compounds detoxification
	Rhodanese domain protein	Cyanide detoxification
	Polysulfide-sulfur transferase Sud (periplasmic)	Cyanide detoxification
Plant growth	Iron binding protein SufA for iron-sulfur cluster assembly	Nitrogen fixation/Biofertilizer
	Quinone	Root nodule and the arbuscular symbionts important for phosphate and nitrogen
	Siroheme	Sulfate and nitrate assimilation
	Riboflavin	Stimulate seed germination and promote seedling development
	Folate	Food fortification
	Molybdenum co-factor	Nitrate assimilation/Biofertilization
	Glutamine synthetase	Nitrate assimilation
	Folate-dependent protein for Fe/S cluster synthesis/repair in oxidative stress	Nitrogen fixation/Biofertiliser
	Molybdenum cofactor biosynthesis protein MoaA	Nitrogen fixation/plant development hormones/Biofertilization
	Fe-S cluster assembly protein SufB	Nitrogen fixation
	Fe-S cluster assembly ATPase SufC	Nitrogen fixation
	Fe-S cluster assembly protein SufD	Nitrogen fixation
	isochorismate synthase DhbC	siderophore synthesis
	TonB-dependent siderophore receptor	siderophore transport
	Ferric siderophore receptor, TonB dependent	siderophore transport
	Siderophore synthetase component, ligase	
	Trilactone hydrolase	
	Bacillibactin synthetase component F	
	Siderophore biosynthesis L-2,4-diaminobutyrate decarboxylase	
	Siderophore biosynthesis protein, monooxygenase	
	Siderophore synthetase component, ligase	

**Fig. 4:**
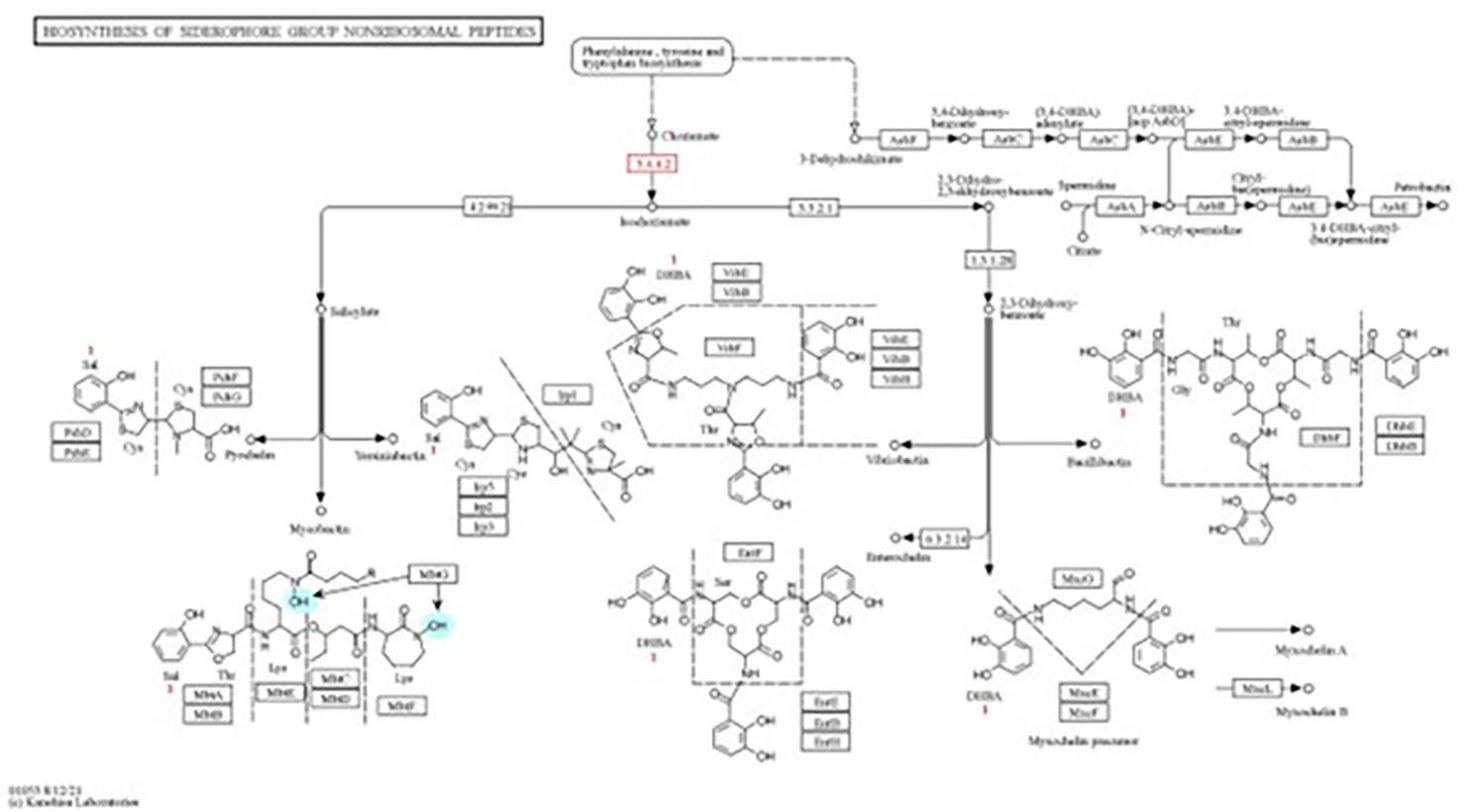
KEGG mapping of genes involved in siderophores synthesis. The elements highlighted in red were identified from the genome of strain MHSD_37.

**Fig. 5:**
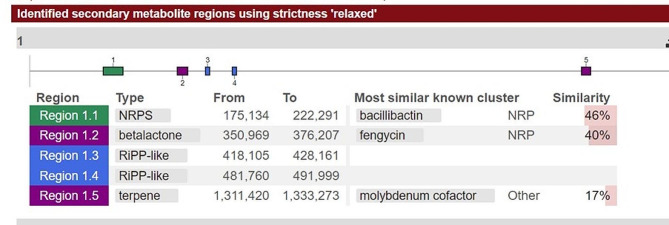
Secondary metabolites identified from MHSD_37 using AntiSmash.

Genes involved in siderophores synthesis and export (Table 4) were identified from the genome of strain MHSD_37. The genes for bacillibactin synthetase component F (*dhb*F), isochorismate synthase (*dhb*C), 2,3-dihydroxybenzoate-AMP ligase (*dhb*E), trilactone hydrolase (*yuiI*) and 2,3-dihydro-2,3-dihydroxybenzoate dehydrogenase, were identified and are involved in the synthesis of the siderophore bacillibactin. Furthermore, isochorismate synthetase and 2,3-dihydro-2,3-dihydroxybenzoate dehydrogenase, were identified in the siderophore biosynthesis pathway, EC 5.4.4.2 and 1.3.1.28, respectively, (Fig. 4). The anti-SMASH analysis also identified a bacillibactin related gene cluster, the non-ribosomal peptide synthetases (NRPSs) (Fig. 5).

Bacterial endophytes also enhance plant growth through the production of phytohormones and vitamins involved in regulation of plant development, cell signalling as well as enhancing nutrient uptake. The strain MHSD_37 genome contains genes that code for vitamins, co-factors, and auxins (Table 4). Genes encoding for the vitamins namely, biotin, thiamine, riboflavin, and folate were identified from strainMHSD_37. Moreover, the presence of genes encoding for the biosynthesis of the auxin, indole acetic acid (IAA), was identified (Table 4).

### Bioremediation

The ability of endophytes to detoxify toxic heavy metals and compounds confers them the advantage to survive in toxic environments. Strain MHSD_37 encodes for several genes that confer resistance to abiotic stress including toxic heavy metals and xenobiotic compounds (Table 4). The strain encodes genes for resistance against heavy metals such as cobalt, zinc, cadmium, copper, and cobalt. The cobalt-zinc-cadmium resistance (*Czc*) family of genes was identified from the strain MHSD_37. The genes encode for proteins which are determinants of the CzcCBA membrane transporters involved in cobalt, zinc, and cadmium resistance.

The genome of MHSD_37 contained a gene encoding for Glutathione S-transferase family protein, which has been reported to play a role in the detoxification of heavy metals and xenobiotic compounds such as 1-chloro-2,4-dinitrobenzene and 7-chloro-4-nitrobenzo-2-oxa-1,3-diazole. Moreover, the genome contained several genes involved in siderophores synthesis and transport (Table 4). The genes involved in the synthesis of siderophores include isochorismate synthase DhbC, siderophore synthetase component, ligase, trilactone hydrolase and bacillibactin synthetase component F. On the other hand, genes that encode for siderophore transport were ferric siderophore receptor, tonB dependent and tonB-dependent siderophore receptor. The genome analysis identified genes that encode for both large and small (monooxygenase and decarboxylase) as well as ligase (Table 4) components of the siderophore synthesis.

### Biocontrol

The strain MHSD_37 genome possesses genes encoding for virulence factors and defence regulators against insect pests and phytopathogens, respectively (Table 4). The genome analysis identified three proteins, HtrA protease/chaperone protein, serine/threonine protein kinases and Kynureninase, which encode for proteins involved in the regulation of defence against phytopathogens. Virulence genes, involved in the infection of pests, were identified from the genome of strain MHSD_37. The strain encodes for the internalin gene responsible for the virulence of insects. The encoded internalin-like protein was reported to have a leucine-rich repeat domain that interacts with host cells, and a C-terminal SLH domain capable of binding to the peptidoglycan layer of the host. Furthermore, the strain contains the gene which encodes for the LSU ribosomal protein L35p, which has roles in host invasion and evasion of intracellular defence.

The genome of strain MHSD_37 contains other genes, involved in host insect virulence and intracellular resistance, such as quinolinate synthetase, osmosensitive K + channel histidine kinase KdpD/E, Glutathione-regulated potassium-efflux system protein, and Trk system potassium uptake protein (Table 4).

### Other potential applications

The genomic analysis of strain MHSD_37 identified the presence of a gene that encodes for the ferritin-like protein 2. Ferritin proteins are widely found in eukaryotes and some bacteria and are composed of 24 subunits forming a cage-like octahedral with a ferroxidase centre. Strain MHSD_37 genome also contains a gene which encodes for the bifunctional enzyme metallophosphatase, with potential application in vaccine industry.

### Annotation of secondary metabolites from the genome of *Bacillus* sp. strain MHSD_37

The anti-SMASH analysis identified the NRPS, betalactone, RiPP, and terpene gene clusters (Fig. 5). The identified gene clusters are involved in the synthesis of natural products with a range of biological activities such as iron acquisition, antimicrobial, antiviral, anticancer, and anti-insecticidal activities.

### Identification and classification of secondary metabolites from *Bacillus* sp. strain MHSD_37

Liquid chromatography–mass spectrometry (LC-MS) analysis identified the presence of biological active secondary metabolites from the excretome of MHSD_37 (Table 5). The strain synthesized and secreted metabolites with anticancer and antimicrobial activity (Table 5). Moreover, a terpene glycoside, Kurilensoside F, with antimicrobial activity was identified from the excretome of strain MHSD_37. The exposure of MHSD_37 to stress, heavy metal Pb, resulted in an increase in the diversity of biological active metabolites identified from the excretome (Table 5). The synthesis and presence of siderophores and surfactants, corneybactin and empigen (Table 5), respectively, is consistent with the bioremediation ability of the strain. A prominent feature was the increase in the number of oligopeptides identified following the exposure of MHSD_37 to Pb.

**Table 5:** Secondary metabolites identified from the secretome of strain MHSD-37 before and after exposure to Pb using LC-MS.

	Precursor (m/z)	RT	Nature of compound	Molecular formula	Compound	Biological activity
**Control**	488.24	6.08	N-acyl amines	C20H33N5O9	Goralatide	Anticancer
	365.28	14.99	Alpha amino acids	C19H39N2O3	Empigen BR	Surfactant
	279.16	16.19	Benzoic acid esters	C16H22O4	Hatcol DBP	Plasticiser
	362.21	19.11	Cinnamic acid esters	C24H27NO2	Octocrylene	Sunscreen
	482.41	19.77	Fatty Acyls	C25H52O7	Tridecylhexaethoxylate	Surfactant
	311.17	19.82	Retro-dihydrochalcones	C20H22O3	Avobenzona	Sunscreen
	427.38	23.65	Fatty alcohol esters	C26H50O4	Witamol 500	Plasticiser
	631.41	24.44	Terpene glycosides	C33H58O11	Kurilensoside f	Antimicrobial
	506.53	24.48	N-acyl amines	C34H67NO	Oleyl palmitamide	Plasticiser
	551.59	24.48	N-acyl amines	C36H74N2O	Butanamide, 4-(dioctylamino)	Anticancer
	547.4	25.53	Benzoic acid esters	C33H54O6	hatcol 2000	Plasticiser
**Pb treated**	734.31	6.13	Diterpenoids	C40H47NO12	3’-N-Debenzoyl-2’-deoxytaxol	Anticancer
	545.26	6.4	Oligopeptide	C22H36N6O10	Acetyl-DTTPA-NH2	Anti-HIV
	261.12	6.73	Alpha amino acid	C14H16N2O3	Maculosin	Antioxidant
	314.17	7	Lipid	C15H22O3	Racemosalactone A	Anticancer
	197.13	7.01	Alpha amino acid	C10H16N2O2	Cyclo(-Pro-Val)	Antifungal
	528.27	7.31	Oligopeptide	C23H37N5O9	n.a.	Antimalarial
	262.14	7.39	Peptide	C14H18N2O3	Phenylalanylproline	Antimcrobial
	530.25	7.61	Oligopeptide	C22H35N5O10	n.a.	Anticancer
	765.34	7.66	Oligopeptide	C38H48N6O11	n.a.	Antimalarial
	408.23	7.69	Oligopeptide	C19H29N5O5	n.a.	Anti-angiotensi II
	680.37	7.69	Oligopeptide	C31H49N7O10	n.a.	Anticancer
	702.35	7.69	Oligopeptide	C36H49N5O8	n.a.	Anti-virus
	401.21	7.76	Oligopeptide	C17H28N4O7	n.a.	Antibacterial
	444.23	7.77	Oligopeptide	C16H29N9O6	n.a.	Anticlots
	888.42	7.79	Oligopeptide	C39H59N11O14	n.a.	Anti-virus
	302.15	7.94	Oligopeptide	C16H19N3O3	D-Proline, D-tryptophyl	Antimicrobial/anticancer
	495.21	8.01	Oligopeptide	C21H28N8O5	n.a.	Antimicrobial/anticancer
	587.31	8.25	Oligopeptide	C25H42N6O10	n.a.	Antimicrobial
	757.32	8.29	Oligopeptide	C39H44N6O10	n.a.	Anticancer
	411.26	8.32	Oligopeptide	C20H34N4O5	n.a.	Antimicrobial
	211.14	8.37	Alpha amino acid	C11H18N2O2	Gancidin W	Antimalarial agent
	578.29	8.39	Oligopeptide	C27H39N5O9	n.a.	Antimalarial agent
	484.25	8.46	alpha amino acid	C26H33N3O6	Carbobenzoxy-Ala-Ile-Phe-COOH	Anti-HIV
	574.32	9.17	Oligopeptide	C29H43N5O7	n.a.	Anti-virus
	701.32	9.29	Phenylalanine	C38H44N4O9	n.a.	Anti-virus
	481.21	9.48	Oligopeptide	C25H28N4O6	n.a.	Anticancer
	883.27	9.88	Cyclic depsipeptides	C39H42N6O18	Corneybactin	Iron acquistion
	365.28	14.78	Alpha amino acids	C19H39N2O3	Empigen BR	Surfactant
	279.16	16.51	Benzoic acid esters	C16H22O4	Hatcol DBP	Plasticiser
	362.21	19.11	Cinnamic acid esters	C24H27NO2	Octocrylene	Sunscreen
	311.17	19.82	Retro-dihydrochalcones	C20H22O3	Avobenzona	Sunscreen
	631.41	24.44	Terpene glycosides	C33H58O11	Kurilensoside f	Antimicrobial
	506.53	24.48	N-acyl amines	C34H67NO	Oleyl palmitamide	Plasticiser
	551.59	24.48	N-acyl amines	C36H74N2O	Butanamide, 4-(dioctylamino)	Anticancer
	547.4	25.53	Benzoic acid esters	C33H54O6	hatcol 2000	Plasticiser

n.a. = no similar annotated compounds in the databases

## Discussion

In this study, *in silico* analysis was used to determine and analyze the biotechnological potential of *Bacillus* sp. strain MHSD_37. The genome *de novo* assembly results showed that strain MHSD_37 had a genome size of 5 139 594 bp and a G + C content of 35.3% which is comparable to other *Bacillus* spp. (Table 1). The TYGS data revealed that MHSD_37 was closely related to *Bacillus albus* strain N35-10-2^T^ (Table 2). However, the observed dDDH was lower than the 70% recommended cutoff points for species delineation [17–18], suggesting that strain MHSD_37 is a novel species. Following a comparison of the dDDH and ANI data, the method of classification for species delineation for strain MHSD_37 was based on the TYGS method for prokaryotic species delineation because the dDDH outperforms the ANI for taxon delineation at the sub and specific level [17]. Therefore, *Bacillus* sp. strain MHSD_37 is a putative novel species based on the dDDH value, and further studies are underway for its taxonomic description and delineation.

### Endophytic lifestyle

The genome annotation identified functional genes involved in evading host defense system, protecting the host from herbivores, and ensuring the availability of limited nutrients which are important characteristic for the endophytic lifestyle. The predicted genes included genes encoding for N-acyl homoserine lactone hydrolase, synthesis of autoinducer-2 (AI-2) and for *Lys*R family transcriptional regulator (Table 3). Zuniga and coworkers [19] demonstrated that N-acyl homoserine lactone hydrolase played an important role in molecular communication and host-endophyte communication. The study by Zuniga and coworkers [19] showed that mutant *Burkholderia phytofirmans* was deficient of N-acyl homoserine lactone-mediated cell-to-cell communication and this subsequently impacted the efficient colonization of *Arabidopsis thaliana* plants. Jiang and coworkers [20] reported that the use of AI-2 inhibitor (Str7410) significantly reduced the formation of multispecies biofilm and subsequently increased their antibiotic susceptibility. The ability of endophytes to target plant host pest and pathogens is beneficial for both the endophyte and host by antagonizing competition and providing protection, respectively [5]. The genes encoding for sensor histidine kinase and two-component sensor kinase *Yvc*Q were also identified from the strain MHSD_37 genome (Table 3). The genes are involved in the early phytopathogen detection [21], and thus play an important role in the protection of the host against pathogens.

### Plant growth promotion

BEs have genes which regulate processes that promote plant growth [22], which can be harnessed for potential biotechnological applications in biofertilization [23]. BEs influence plant growth promotion through mechanisms such as phytohormone modulation, improving plant nutrient availability or uptake, as well as enhancing plant tolerance to stress and toxic heavy metals or hydrocarbons [5]. The role of the Fe-S cluster, encoded by the strain MHSD_37 (Table 4), is electron transfer to the nitrogen fixation (NIF) regulators [24–26], and thus plays a crucial role in nitrogen fixation. Moreover, the NIF system is responsible for the maturation of nitrogenase [24], an enzyme responsible for nitrogen fixation [27]. Amino acid substitutions at C- and N-terminal domains of a *Azotobacter vinelandii* NifU protein reportedly resulted in the deficiency of nitrogenase-specific [Fe-S] cluster formation [28].

Phosphorus is an essential plant micronutrient involved in many physiological processes. Although there is an abundance of phosphorus in the soil, it is available in the form of insoluble phosphates. The biosynthetic genes for exopolyphosphatase *(ppx*), secreted alkaline phosphatase (*sap*), and alkaline phosphatase synthesis transcriptional regulatory protein (*pho*P) (Table 4), play a significant role in phosphate solubilization. Therefore, bacterial endophytes play a crucial role in the solubilization of phosphate making it easily available for their host and consequently supporting plant development and growth. Singh and Arora [29] reported that the use of the phosphate solubilizing endophyte *Pseudomonas* sp., as bioinoculant, significantly enhanced the growth and yield of the medicinal plant *Withania somnifera* under a nutrient-liming saline environment. Moreover, the presence of alkaline phosphatase was also detected from the soil following the bio-inoculation.

Endophytic bacteria play a crucial role in iron acquisition for their plant host, under iron limiting conditions. As such genes involved in siderophores synthesis and export (Table 4) were identified from the genome of strain MHSD_37. Endophytes synthesize iron chelating siderophores capable of binding to insoluble ferric ions and subsequently deliver them to the plant through root-based ligand exchange [14]. Radziki and coworkers [30] demonstrated that the treatment of tomato plants, cultured in hydroponics, with siderophore producing *Chryseobacterium* sp. C138 was adequate to deliver iron to plants through the roots which significantly improved the yields. The bio-inoculation of *B. subtilis*-LSBS2 and application of pure siderophore solution to the sesame plants significantly increased the iron content in the plants by 47 and 19%, respectively, compared to the control treatment [7].

Furthermore, the vitamins, encoded by the genes identified from strain MHSD_37 (Table 4), promote and enhance plant root colonization by arbuscular mycorrhiza important for nitrate and sulfate assimilation [31–32]. In addition, the genome analysis also identified genes encoding for the co-factors, siroheme and molybdenum (Table 4), involved in nitrate and sulfate assimilation [26, 33]. Indole acetic acid (IAA) genes, a predominant plant auxin involved in cell signalling, plant growth regulation and the induction of plant defence [34], were also identified. IAA is not only involved in physiological process but is involved in the regulation of the synthesis of other plants hormones such as ethylene [35], which regulates a plant’s response to abiotic and biotic stress [36].

### Bioremediation

The ability of endophytes to detoxify toxic heavy metals and compounds confers them the advantage to survive in toxic environments. Furthermore, the presence of genes for and mechanisms of heavy metals and organic compounds detoxification offers an opportunity for the exploration of their potential in the remediation of waste or contaminated water and soil. Bioremediation is a cheaper and environmentally friendly alternative method for the remediation of contaminated water and soil. The *Czc* genes identified from strain MHSD_37 (Table 4), encodes for the CzcCBA pump which uses an ion efflux driven mechanism to remove toxic heavy metals from the cell cytoplasm [37]. Cabral and colleagues [38] reported the presence and increased transcription of *Czc* genes in *Desulfobacterium autotrophicum* growing in oil contaminated soil with a high concentration of Zn, Pb and Cu.

An important group of functional genes identified from the genome of strain MHSD_37 encodes for the synthesis of siderophores (Tables 3 and 5). In addition to their role in iron assimilation, for the host plant, siderophores are capable of chelating and forming complexes with other heavy metals and metalloids [39]. Giovanella and coworkers [40] reported that the production of siderophores was important for the removal of Cd and Pb using *Pseudomonas* sp. B50D, which resulted in 60 and 85% removal, respectively. *Bacillus amyloliquefaciens* NAR38.1 reported a significant increase in the extracellular production of siderophores when grown under Pd and Al contaminated conditions [12]. Siderophores are also involved in the remediation of organic contaminants such as 2-chlorphenol, 4-chlorphenol, 4-Cl-nitrobenzene, pyrene, and hydrocarbons [7]. Siderophores are involved in the remediation of the organic contaminants directly through the induction of RO species production or indirectly through enhancing the bioavailability of the contaminants [41].

### Biocontrol

The interaction between bacterial endophytes and their host is characterized by the protection of the latter from phytopathogen and herbivores. This is of relevance to the agricultural sector and thereby makes endophytes a potential source for the prospection of bioproducts for applications in pest control [42–43]. Millan and coworkers [43] reported the upregulation of the serine/threonine protein kinases and kynureninase genes, which are also coded by strain MHSD_37, in *Metschnikowia pulcherrima* in the presence of *B. cinerea* spores in wounded apples. The genes are involved in signal transduction and the biosynthesis of cofactors as well as secondary metabolites, respectively.

The strain encodes for the internalin gene (Table 4) responsible for the virulence in insects. The internalin genes were reportedly induced following the inoculation and oral infection of the insect *Galleria mellonella* by *Bacillus cereus* [44]. The encoded internalin-like protein was reported to have leucine-rich repeat domain that interacts with host cells, and a C-terminal SLH domain capable of binding to the peptidoglycan layer of the host [43]. Furthermore, the strain contains the gene which encodes for the LSU ribosomal protein L35p (Table 4), which has roles in host invasion and evasion of intracellular defence [45].

Quinolinate synthetase and KdpD/E histidine kinase genes were also identified from the genome of the strain (Table 4). Quinolinate synthetase plays a role in the virulence of bacterial symbionts associated with entomopathogenic nematodes [46–47]. KdpD/E histidine kinase is a transcriptional regulator of genes involved in virulence [48]. Moreover, KdpD/E is responsible for osmotic, oxidative, and antimicrobial stress. Alegado and coworkers [49] illustrated that KdpD/E mutant *Salmonella typhimurium* was defective of persistent and survival in the nematode *Caenorhabditis elegans*.

### Other biotechnological applications

The genome of strain MHSD_37 has genes which encodes for ferritin protein and 5’-nucleotidase (Table 4). Ferritin proteins have potential bionanotechnology applications such as biomineralization [50], drug delivery [51], and medical imaging [52]. For instance, the iron oxide and hydroxides of ferritins have good superparamagnetic properties thus making the proteins efficient contrast agents applicable in magnetic resonance imaging (MRI) [53]. 5’-nucleotidase have a broad substrate specificity and catalyse the hydrolytic dephosphorylation of 5′-ribonucleotides and 5′-deoxyribonucleotides to their respective nucleosides and phosphate [54]. Nucleotidase are also involved in cell-to-cell communication, nucleic acid repair, and signal transduction, and control of the ribo- and deoxyribonucleotide pools [55]. Therefore, the prevalence of cell wall anchored 5’-nucleotidase in most human bacterial pathogens as well as such roles in cell-to-cell communication and signal transduction [56], make nucleotidase potential targets for vaccine development.

### Secondary metabolites from *Bacillus* sp. strain MHSD_37

The strain synthesized and secreted metabolites with anticancer and antimicrobial activity (Table 5). Li and coworkers [57] synthesized a goralatide analog with selective anti-leukemic activity against human myeloid HL-60, HEL, Nalm-6 leukemia cells, endothelial HUVEC, glioblastoma U251 and transformed kidney 293T cells. Moreover, a terpene glycoside, Kurilensoside F, with antimicrobial activity [58] was identified from the excretome of strain MHSD_37. The synthesis and presence of siderophores and surfactants, corneybactin and empigen (Table 5), respectively is consistent with the bioremediation ability of the strain. Bacterial surfactants have been reported to form complexes with heavy metals thereby improving their solubility and bioavailability for further detoxification [59].

## Conclusions

*Bacillus* sp. strain MHSD_37 has potential for biotechnological applications in bioremediation, biocontrol, and biofertilization. The strain also possesses genes encoding for bioproducts with potential application in biocontrol, biofertilization, and bioremediation. The genome also contains genes which potential novel application in bio-nanotechnology and vaccine development. Moreover, LC-MS data identified the presence of biologically active metabolites further confirming the biotechnological potential of strain MHSD_37. The current analysis and data lay a foundation for further development of strain MHSD_37 for application in the different biotechnologies identified. Future studies will entail the development of bioprocessing methods for the large-scale production of endophytes or their products and characterization of their efficacy in field trials. Moreover, the development of recombinant production systems for protein required in bio-nanotechnology and vaccine development will play an important role in the exploitation of this strain for nanotechnology and pharmaceutical applications.

### Methods

#### Bacterial strains maintenance and growth

The bacterial strains were isolated from sterilized leaves of the medicinal plant *S. nigrum*, according to the method of Mahlangu and Serepa-Dlamini [60]. A 30% glycerol stock of the bacterial cultures were plated on nutrient agar (NA) plates and incubated for 24 h at 28 °C, for routine culture maintenance. The bacteria were further grown on nutrient broth (NB) at 28 °C, agitating at 150 rpm for 24 h.

#### Genomic deoxyribonucleic acid isolation, library preparation, and sequencing

Genomic DNA was extracted from solid colonies using the NucleoSpin microbial DNA extraction kit according to the manufacturer’s protocol (Macherey-Nagel, Germany). The DNA was sequenced at a commercial service provider, Biotechnology Platform, Agricultural Research Council, Onderstepoort, South Africa. Paired-end libraries (2 × 150 bp) were generated using the NextEra DNA sample preparation kit (Illumina, United States), and sequencing was performed on the HiSeq 2,500 platform.

#### Genome assembly and annotation

The genome quality control, trimming, and assembly were performed on GALAXY accessible from https://usegalaxy.org/ [61]. The FastQC (version 0.72.0) [62] was used for quality control of the raw sequence reads followed by trimming with the Trimmomatic (version 0.38.0) [63]. The sequence reads were *de novo* assembled using Unicycler (version 0.4.8.0) [64], and the quality was assessed with Quast (Galaxy Version 5.0.2) [65]. The draft genome was annotated using the National Center for Biotechnology Information—Prokaryotic Genome Annotation Pipeline [66] and Rapid Annotations using Subsystems Technology [67]. The presence of Clustered Regularly Interspaced Short Palindromic Repeats (CRISPR) sequences and phages was checked with CRISPRFinder [68] and PHASTER [69], respectively.

#### Phylogenome analysis

A whole genome-based taxonomic analysis was performed from the free bioinformatics platform, Type (Strain) Genome Server (TYGS), accessible from; https://tygs.dsmz.de [17]. The pairwise comparisons among the set of genomes were performed with the Genome Blast Distance Phylogeny and accurate intergenomic distances inferred under the algorithm trimming and distance formula *d2*. The average nucleotide identity (ANI) values between the strain and closely related species were calculated with Orthologous Average Nucleotide Identity (OrthoANI) tool [70].

#### Liquid chromatography–mass spectrometry analysis

The bacterial excretome, following exposure to lead (Pb), was analyzed with a liquid chromatography-quadrupole time-of-flight tandem mass spectrometer (LC–MS-9030 q-TOF, Shimadzu Corporation, Kyoto, Japan) fitted with a Shim-pack Velox C18 column (100 mm × 2.1 mm with particle size of 2.7 m). The column oven temperature was maintained at 50 °C. The injection volume was 5 µL, and the samples were analytically separated over a 30 min binary gradient. A constant flow rate of 0.04 mL/min was applied using a binary solvent mixture of water with 0.1% formic acid and 0.1% formic acid in acetonitrile. The gradient technique was gradually increased from 3 to 30 min to facilitate the separation of the compounds within the samples. Eluent B was kept at 5% from 0 to 3 min, gradually increased from 5 to 40% between 3 and 5 min, and finally increased to 40–95% between 5- and 23-min. Eluent B was subsequently kept isocratic at 95% between 23 and 25 min. The gradient was returned to original conditions of 5% at 25–27 min, and re-equilibration at 5% occurred at 27–30 min. The liquid chromatographic eluents were subsequently subjected to a Quadruple Time-of-Flight high-definition mass spectrometer for analysis in positive electrospray ionization (ESI) mode with the following conditions: 400 °C heat block temperature, 250 °C desolvation line (DL) temperature, 42 °C flight tube temperature, and 3 L/min nebulization and dry gas flow. The data was acquired using the data-dependent acquisition (DDA) mode, which simultaneously generated MS1 and MS2 data for all ions within a mass-to-charge ratio (m/z) range of 100–1500 (precursor m/z isolation window) and an intensity threshold above 5000. The MS2 Experiments were conducted utilizing argon gas as the collision gas and a collision energy of 35 eV with a spread of 5 and sodium iodide as a calibration solution to monitor high mass precision. Metabolite annotation was completed at Metabolomics Standards Initiative (MSI) levels 2 and 3. The former is based on the retention time, mass-to-charge ratio (m/z), and fragmentation patterns matching data available from the databases in Sirius [71–73]. The fragments with no matches to anything on the databases were classified according to their compound class according to the molecular networking from Canopus on Sirius [72, 73].

## Data Availability

The data from this Whole Genome Shotgun project has been deposited at DDBJ/ENA/GenBank under the accession JAVIVK000000000, BioSample accession number SAMN36845528, and BioProject accession number BioProject ID: SAMN37198868. The version described in this paper is JAVIVK000000000.

## Abbreviations

Bes: Bacterial endophytes
IAA: Indole acetic acid
ACC: 1-aminocyclopropane-1-carboxylate deaminase (ACC)
WGS: Whole genome analysis
CDS: Coding sequence
ANI: Average nucleotide identity
dDDH: Digital DNA–DNA hybridization
QS: Quorum sensing
ROS: Reactive oxygen species
EPS: Exopolysaccharide
LC-MS: Liquid chromatography–mass spectrometry
NIF: Nitrogen fixation
NA: Nutrient agar
